# Prognostic and predictive value of radiomics features at MRI in nasopharyngeal carcinoma

**DOI:** 10.1007/s12672-021-00460-3

**Published:** 2021-12-17

**Authors:** Dan Bao, Yanfeng Zhao, Zhou Liu, Hongxia Zhong, Yayuan Geng, Meng Lin, Lin Li, Xinming Zhao, Dehong Luo

**Affiliations:** 1 Department of Radiology, National Cancer Center/National Clinical Research Center for Cancer/Cancer Hospital, Chinese Academy of Medical Sciences and Peking Union Medical College, Beijing, 100021 China; 2 Department of Radiology, National Cancer Center/National Clinical Research Center for Cancer/Cancer Hospital & Shenzhen Hospital, Chinese Academy of Medical Sciences and Peking Union Medical College, Shenzhen, 518116 China; 3 Huiying Medical Technology (Beijing) Co., Ltd, HaiDian District, B-2 Building, Dongsheng Science Park, Beijing City, 100192 People’s Republic of China

**Keywords:** Nasopharyngeal carcinoma, Radiomics, Magnetic resonance imaging, Disease progression, LASSO Cox regression analysis

## Abstract

**Purpose:**

To explore the value of MRI-based radiomics features in predicting risk in disease progression for nasopharyngeal carcinoma (NPC).

**Methods:**

199 patients confirmed with NPC were retrospectively included and then divided into training and validation set using a hold-out validation (159: 40). Discriminative radiomic features were selected with a Wilcoxon signed-rank test from tumors and normal masticatory muscles of 37 NPC patients. LASSO Cox regression and Pearson correlation analysis were applied to further confirm the differential expression of the radiomic features in the training set. Using the multiple Cox regression model, we built a radiomic feature-based classifier, Rad-Score. The prognostic and predictive performance of Rad-Score was validated in the validation cohort and illustrated in all included 199 patients.

**Results:**

We identified 1832 differentially expressed radiomic features between tumors and normal tissue. Rad-Score was built based on one radiomic feature: CET1-w_wavelet.LLH_GLDM_Dependence-Entropy. Rad-Score showed a satisfactory performance to predict disease progression in NPC with an area under the curve (AUC) of 0.604, 0.732, 0.626 in the training, validation, and the combined cohort (all 199 patients included) respectively. Rad-Score improved risk stratification, and disease progression-free survival was significantly different between these groups in every cohort of patients (p = 0.044 or p < 0.01). Combining radiomics and clinical features, higher AUC was achieved of the prediction of 3-year disease progression-free survival (PFS) (AUC, 0.78) and 5-year disease PFS (AUC, 0.73), although there was no statistical difference.

**Conclusion:**

The radiomics classifier, Rad-Score, was proven useful for pretreatment prognosis prediction and showed potential in risk stratification for NPC.

**Supplementary Information:**

The online version contains supplementary material available at 10.1007/s12672-021-00460-3.

## Introduction

Nasopharyngeal carcinoma (NPC) is the most common tumor occurring in the nasopharynx. As reported, there were approximately 130,000 new cases of NPC and 73,000 NPC-related deaths worldwide in 2018 [1]. Despite the proven effectiveness of radiotherapy and concurrent or adjuvant chemotherapy, about 30–40% of NPC patients still suffer from disease relapse after initial control [2]. It’s extremely challenging to deal with relapsed NPC including loco-regional recurrence and distant metastasis, with death caused by distant metastasis alone can be as high as 62% [3]. Therefore, how to accurately stratify the risk of disease relapse in advance and individualize the treatment plan accordingly is highly desired in the new era of precision medicine, which would greatly further improve the overall prognosis.

With superb soft-tissue contrast and no radiation, MRI has been integrated into the whole workflow of NPC management, including lesion detection and diagnosis, TNM staging, radiotherapy guiding, treatment response evaluation, etc. With the increasing amount of MR image data accumulating, now it was realized that certain information contained in MR image, i.e. intra-lesion heterogeneity was under-utilized in the past, which should be further exploited. With the advent of a new age of big data mining, radiomics emerges as a brand-new way to non-invasively and quantitatively translate lesion spatial heterogeneity into high-dimensional image features that used to be nearly impossible to be quantified by our bare eyes and thus could provide additional valuable evidence to improve decision-making in cancer management [4]. Several studies have attempted to investigate the potential clinical relevance of radiomics features and their value in predicting treatment response, clinical staging, survival and prognosis, prediction in NPC [5, 6]. Although the favorable value of radiomics analysis in managing NPC has been reported in some preliminary studies [7, 8], its value could be further confirmed before it could be fully translated into the clinical workflow of managing NPC. In this study, we tried to find out the radiomics features that were different between the primary lesion of NPC and normal tissues (e.g., normal masticatory muscles). We hypothesized that radiomic features from pretreatment MRI were associated with and could yield accurate prediction of the disease progression in patients with NPC.

Hence, in this study, we aimed to investigate the performance of a radiomics model in predicting disease progression-free survival for patients with non-metastatic NPC after tumor complete remission.

## Materials and methods

### Patients and clinical characteristics

**Fig. 1:**
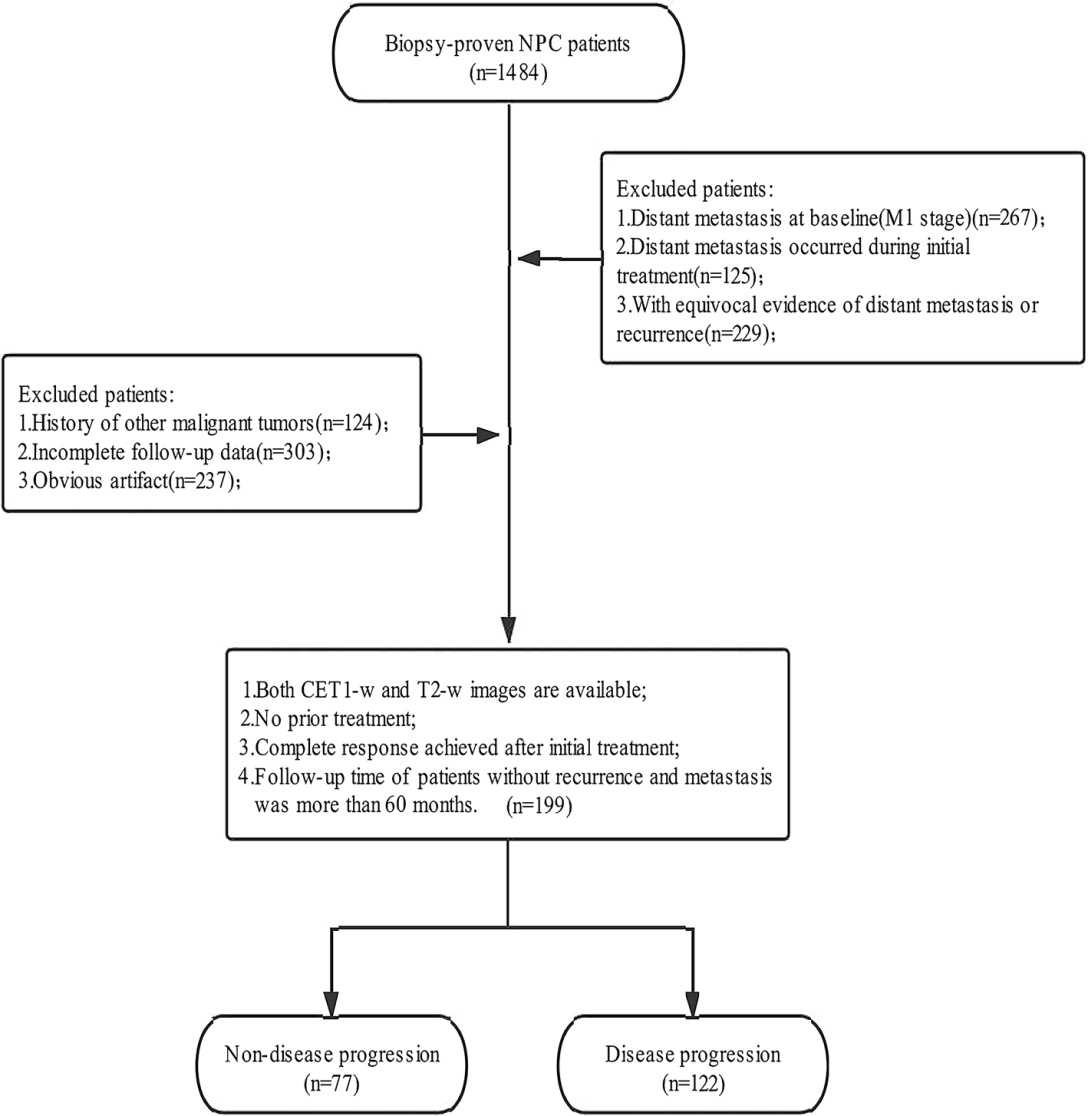
Flowchart of included pathway for patients

This retrospective study was approved by the institutional review board and the requirement for written informed consent from each patient was waived. Between January 2011 and December 2018, 1484 consecutive patients with pathologically confirmed NPC at our institution were retrospectively included. We only included those patients: (a) who underwent nasopharynx-neck MRI within 2 weeks before any type of anti-tumor treatment; (b) with no apparent artifacts of any type potentially affecting imaging analysis; (c) without evidence suggesting distant metastasis in the baseline assessment; (d) who achieved complete response after initial treatment; (e) who were then followed up for at least 60 months after complete response. As a result, a total of 199 patients were included in this study. With hold-out cross validation scheme (training set: validation set = 8:2, the number of random seed = 867), they were randomly allocated to a training set (159 patients) and a validation set (40 patients) (Fig. 1). Patient characteristics were summarized in Table 1.

**Table 1:** Baseline clinical characteristics of the patients

Clinical characteristic	All patients	Training set	Validation set	P value
Age (years old)				0.861
< 40	67	54	13	
≥ 40	132	105	27	
Gender				0.518
Male	152	123	29	
Female	47	36	11	
Histology				0.860
Differentiated	97	78	19	
Undifferentiated	102	81	21	
EGFR				0.749
Positive	171	136	35	
Negative	28	23	5	
VEGF				0.782
Positive	43	35	8	
Negative	156	124	32	
T stage				0.503
T1	22	19	3	
T2	30	24	6	
T3	95	72	23	
T4	52	44	8	
N stage				0.200
N0	19	16	3	
N1	48	42	6	
N2	89	71	18	
N3	43	30	13	
Overall stage				0.348
II	20	18	2	
II	89	72	17	
IV	90	69	21	
Treatment				0.403
R	27	24	3	
CC	69	56	13	
RT	16	13	3	
CCT	37	31	6	
ICC	33	24	9	
ICT	17	11	6	
Death				0.304
Disease progression	17 (8.5%)	16 (94.1%)	1 (5.9%)	
Accident	1 (0.5%)	1 (100%)	0	
Unknown^a^	22 (11.1%)	15 (68.2%)	7 (31.8%)	
Local–regional recurrence^b^	49 (24.6%)	39 (79.6%)	10 (20.4%)	0.951
Distant metastasis^b^	77 (38.7%)	58 (75.3%)	19 (24.7%)	0.201
Censored	72 (36.2%)	61 (84.7%)	11 (15.3%)	0.201

^a^Of the 22 patients with unknown cause of death, 18 have already developed local–regional recurrence or distant metastasis in the follow-up in our hospital. ^b^4 patients developed local–regional recurrence and distant metastasis. *EGFR* epidermal growth factor receptor, *VEGF* vascular endothelial growth factor, *R* Radiotherapy, *CC* Concurrent Chemoradiotherapy, *RT* Radiotherapy + Targeted therapy, *CCT* Concurrent Chemoradiotherapy + Targeted therapy, *ICC* Induction Chemotherapy + Concurrent Chemoradiotherapy, *ICT* Induction Chemotherapy + Concurrent Chemoradiotherapy + Targeted therapy

From the medical records, clinical characteristics were collected including age, gender, histopathology, T-stage, N-stage, overall clinical stage, treatment regimen, epidermal growth factor receptor (EGFR), and vascular endothelial growth factor (VEGF) mutation status. Tumor staging was initially performed by Y.Z. (with 17 years of experience in head and neck imaging) and then reviewed by D.L. with 31 years of experience specialized in head and neck imaging according to the TNM classification (8th edition American Joint Committee on Cancer/Union for International Cancer Control) [9], with any disagreement resolved through discussion.

### Follow-up surveillance

Three months after the completion of initial treatment, treatment response was evaluated for each patient. Only those who achieved complete response on MRI and confirmed by nasopharyngoscopy examination with biopsy were included. Included patients were then followed up at least every 3 months during the first 2 years and every 6 months starting from the third year. The data were censored on December 31, 2020. Disease progression-free survival (PFS) was defined as the time from the date of complete response confirmation to the date of the first development of local–regional recurrence, distant metastasis, death from any cause, or last follow-up.

### MR technique

All of the MR images were acquired using 3.0T MR scanners (GE Discovery MR 750, General Electric Medical Systems, US) with an 8-channel head and neck phased array coil. Axial FSPGR contrast-enhanced T1-weighted imaging (CE-T1WI) was performed 60 s after intravenous bolus injection of gadopentetate dimeglumine (Magnevist, Bayer, Leverkusen, Germany) at a dosage of 0.2 ml/kg of body weight and 1.5 ml/second using a power injector. Imaging protocol with parameters used is detailed in Additional file 1.

### MR-based radiomics analysis

**Fig. 2:**
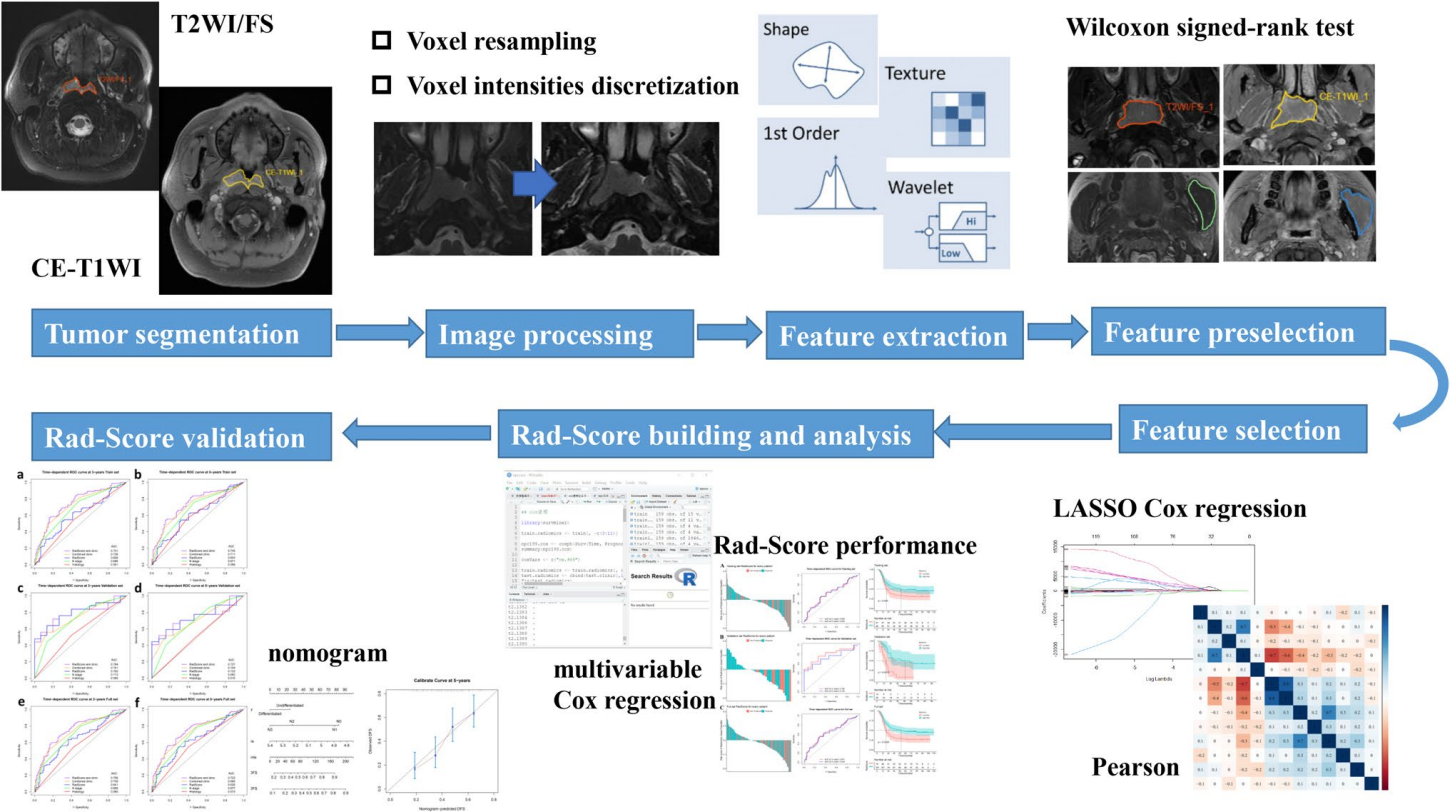
Workflow of the radiomics analysis

The radiomics workflow is shown in Fig. 2.

### Image segmentation

On the radiomics cloud platform V2.1.2, tumor and normal tissue regions of interest (ROI) were manually segmented on the T2-weighted fat suppression images (T2WI/FS) and contrast-enhanced T1-weighted images (CE-T1WI) initially slice by slice by reader 1 (D.B. with 4 years of experience in head and neck imaging) and then reviewed by reader 2 (D.L.). To generate radiomic features that were differentially expressed between tumors and normal tissue, we randomly selected 37 patients from the 159 patients with NPC included in the training set and delineated the normal tissue ROI on one slice (the largest level) of the masticatory muscle (masseter muscle) on the left side of the patient. We originally planned to randomly select 40 patients (25%). However, due to platform failure and some other reasons, we could only export the radiomics features of 37 patients from the platform at this stage. For each tumor lesion, the 3D volumes of interests (VOIs) were obtained by stacking each ROI on the 2D consecutive slice to cover the whole NPC tumor (Additional file 2). To test the reproducibility, tumor VOIs of 30 randomly chosen patients were repeatedly segmented by reader 3 (Y.Z) within an interval of 1 month. The Dice similarity coefficient (DSC) for an absolute agreement was calculated between pre- and post-segmented VOIs. DSC of < 0.6, 0.6–0.8, 0.8–1.0, and 1.0 indicates inadequate, good, very good, and ideal consistency, respectively [10].

### Radiomics feature extraction

Image preprocessing and feature extraction were performed on the radiomics cloud platform V2.1.2 (Huiying Medical Technology Co., Ltd., the Python software, version 3.7.1; Pyradiomics package, version2.12) (Additional file 3). To ensure spatial consistency in texture analysis across all the images, all images were normalized by centering to the mean standard deviation, resampled to a voxel size of 1 × 1 × 1 mm^3^ using B-Spline interpolation with gray-level discretized by a fixed bin width of 25 in the histogram.

A total of 1409 features were extracted, and they were classified into four categories according to the feature calculation method: (1) shape characteristic (14); (2) first-order statistical characteristics (18); (3) texture features (75), including Gray Level Co-occurrence Matrix, Gray Level Dependence Matrix, Gray Level Run Length Matrix, Gray Level Size Zone Matrix, and Neighborhood Gray Tone Difference Matrix; (4) high-order statistical characteristics (1302), which were based on the mathematical transformation or filter, including logarithm, exponential, gradient, square, squareroot, lbp-2D, and wavelet (Additional file 4).

### Feature selection

Radiomics features were extracted from tumors and normal masticatory muscles of 37 patients on the T2WI/FS and CE-T1W images, respectively. Based on the fact that the possible influence on the analysis of differential expression features caused by the separate radiomics features extraction on 3D tumor volume and on 2D masticatory muscle is acceptable [11, 12]. We performed the Wilcoxon signed-rank test [13] with P < 0.01, on all but 28 shape-related features for the purpose to select features, respectively. The shape-related radiomics features of the primary NPC lesions of all patients were then incorporated into subsequent radiomics analysis together with the selected differentially expressed radiomics features between tumors and normal tissue. Based on the Wilcoxon signed-rank test results, we further validated NPC-associated radiomic features in the training set to assess and validate the prognostic value of every candidate radiomic feature.

The least absolute shrinkage and selection operator (LASSO) Cox regression algorithm [14, 15] was used to reduce irrelevant features on the training set, including the shape-related radiomics features of the primary NPC lesions and the selected differentially expressed radiomics features between tumors and normal tissue. Next, Pearson correlation analysis was performed to further reduce the redundancy of radiomic features with one of the paired significantly correlated features removed (P < 0.05, and correlation coefficient > 0.2) [16]. Which significant/highly correlated features were removed or retained from a given pair of features was unknown, but completely random. The multivariable Cox regression analysis was finally applied to select the most relevant radiomics features with disease progression to avoid overfitting of the model performance. We used multivariable Cox regression analysis to select the most useful prognostic markers among Rad-Score and clinical features, with P < 0.05 indicating an independent significant variable.

### Model development and validation

The most relevant radiomics features with disease progression were used to build a radiomics model called Rad-Score for predicting the disease PFS of NPC patients in the training set. The optimal cutoff of the Rad-Score was determined from the training cohort by the “survminer” package in R software (Additional file 5). To assess the performance of the radiomics prognostic model, the Kaplan–Meier estimator curves and the area under the curve (AUC) comparing the predicted and observed PFS were calculated.

The selected independent significant clinical variables were combined for the final classification modeling by multivariable Cox regression analysis. We investigated the performance of each clinical feature and Rad-Score in predicting prognosis using time-dependent receiver operating characteristic (ROC) analysis at different times being used to measure predictive accuracy with package “timeROC” in R (Additional file 6).

To provide a more understandable outcome measure, a nomogram was then constructed. Calibration curves were plotted via bootstrapping with 1000 resamples to assess the calibration of the radiomics model. Decision curve analysis (DCA) was used to calculate the net benefit from the use of different models at different threshold probabilities in the validation dataset. Patients were classified into high-risk or low-risk groups according to the radiomics model.

### Statistical analysis

Independent samples student t-test or the Mann–Whitney U test was used for comparing continuous variables, while chi-squared (χ^2^) test or Fisher's exact test was for categorical variables. For survival analyses, the correlation between variables and disease PFS was estimated using the Kaplan–Meier method and compared using the log-rank test. Cox regression model was used to do the multivariable survival analysis, and Cox regression coefficients to generate nomograms. Calibration plots were generated to explore the performance characteristics of the nomograms. Statistical analysis was performed with R software (version 4.0.5, http://www.r-project.org); R packages are listed in Additional file 7. R codes are available at GitHub (https://github.com/baodanhb/Nasopharyngeal-carcinoma-and-radiomics.git). P < 0.05 was indicative of a statistically significant difference.

## Results

### Characteristics of the study cohorts

Baseline characteristics of 199 NPC patients are summarized in Table 1. Clinicopathologic characteristics did not differ between the training and validation cohorts. The median follow-up was 26.9 months (interquartile range, 14.2–79.3 months). During the follow-up period, 122 patients developed local–regional recurrence or distant metastasis within the median follow-up duration of 15.6 months (range, 10.9–24.5 months).

### Interobserver variability between readers

Segmentations at two readers showed very good agreement in the manual segmentation with a Dice value of 0.85 ± 0.03 (range 0.78–0.91) for T2WI/FS sequences and 0.84 ± 0.04 (range 0.77–0.92) for CE-T1WI sequences.

### Radiomic feature selection and Rad-Score construction

Among all 2818 radiomic features extracted from T2WI/FS and CET1WI images, we identified 1846 radiomics features including features differentially expressed between tumors and normal tissue and shape-related features. Using the LASSO regression algorithm, based on the differentially expressed radiomic features, 128 radiomic features were selected in the training cohort. Then, a Pearson correlation analysis was then calculated from the above features to detect highly collinear radiomics features. After eliminating significant highly correlated features (defined as P < 0.05 and Pearson's r > 0.2), four radiomics features were retained for the subsequent analysis.

One most relevant disease progression-related radiomic feature, CE-T1WI_wavelet.LLH_GLDM_Dependence-Entropy was selected by the multivariable Cox regression analysis (Table 2). Based on this radiomic feature, we derived a Rad-Score in the training set. The Rad-Score indicated a favorable prediction of 5-year NPC disease progression with an AUC of 0.604 in the training cohort, 0.732 in the validation cohort, and 0.626 in the combined cohort (all 199 patients included).

**Table 2:** Multivariable Cox regression analysis of four radiomic features of disease progression in the training cohort

Variable	β	SE	z	P	HR	95%CI	
						lower	upper
First-order	− 0.0209	0.458	− 0.05	0.96	0.979	0.399	2.40
Shape	0.00199	0.0196	0.10	0.92	1.00	0.964	1.04
GLCM	47.0	341	1.38	0.17	2.64e+20	2.59e−09	2.70e+49
GLDM	269	1.36	1.97	0.04^*^	1.48	1.02	214

*SE* standard error, *HR* hazard ratio, *CI* confidence interval, *First-order* CET1-w_original_firstorder_Median, *Shape* CET1-w_original_shape_LeastAxisLength, *GLCM* CET1-w_original_GLCM_JointAverage, *GLDM* CET1-w_wavelet.LLH_GLDM_DependenceEntropy. * indicates significant difference

### Prediction model development and validation

**Fig. 3:**
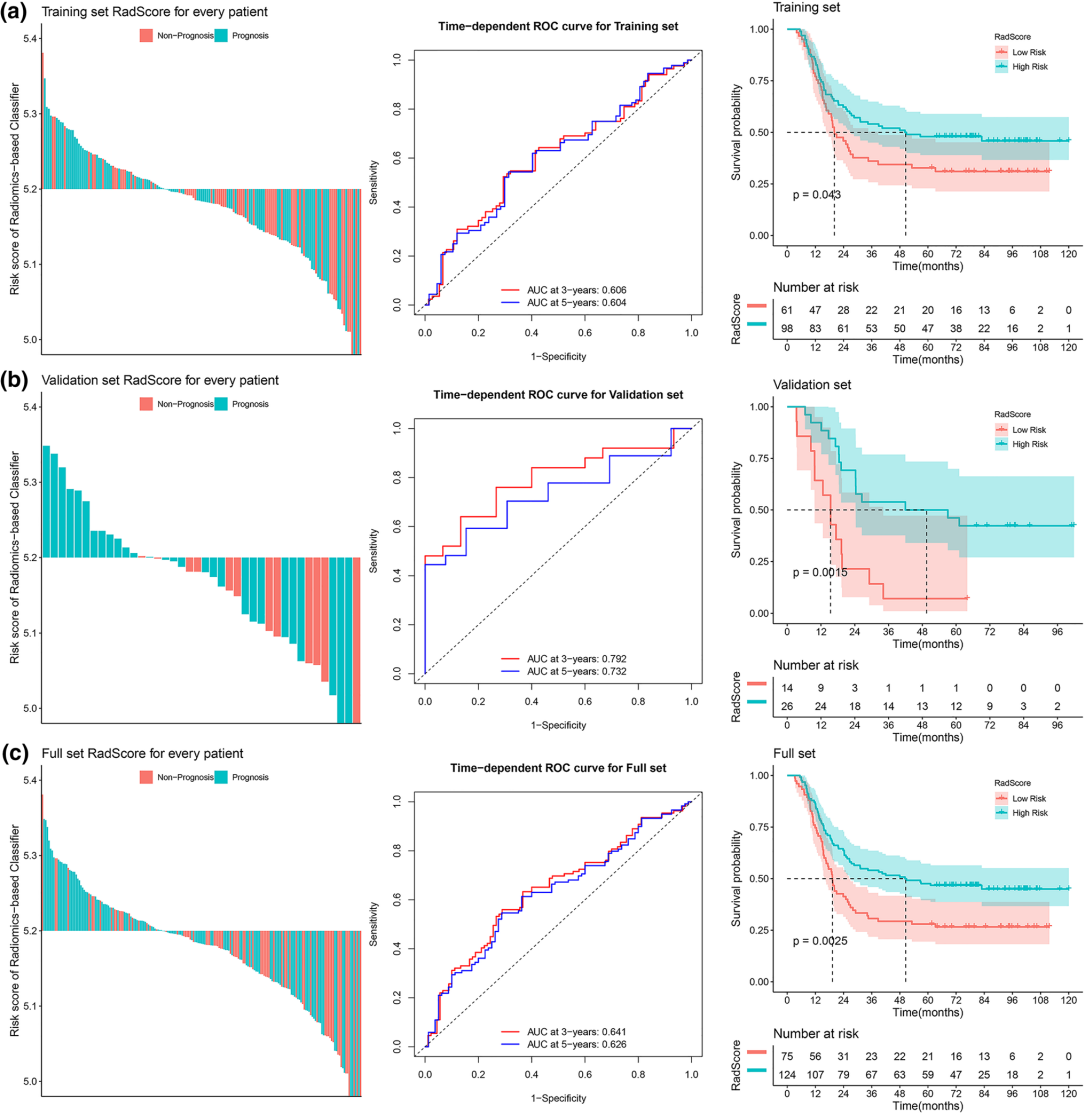
Risk score by the Rad-Score, time-dependent ROC curves and Kaplan–Meier survival in the training, validation, and whole NPC patients sets. **a** Training cohort. **b** Validation cohort. **c** Combined cohort

The optimum cutoff value of the Rad-Score was 5.2 by the COX regression analysis from the training cohort. Based on the optimum cutoff value, we included those patients with a higher radiomics value (≥ 5.2) in the group of patients at high risk of disease progression (high-risk group), and those with a lower radiomics value (< 5.2) at low risk of disease progression (low-risk group). When we assessed the distribution of risk value and survival status, patients with lower risk value generally had better survival than did those with higher risk value (Fig. 3a, left panel). We assessed the prognostic accuracy of the radiomic feature-based classifier with time-dependent ROC analysis at varying follow-up times (Fig. 3). 5-year disease PFS was 32.8% (95% confidence interval [95% CI] 22.9–47.0) for the high-risk group, and 48.0% (39.0–59.0) for the low-risk group (hazard ratio [HR] 1.52, 95% CI 1.01–2.28; P = 0.044; Fig. 3a).

We did the same analyses with 40 NPC patients from the validation cohort. 5-year disease progression-free survival was 7.1% (95% CI 1.1–47.2) for the high-risk group and 46.2% (30.5–70.0) for the low-risk group (HR 3.28, 95% CI 1.52–7.07; P < 0.002; Fig. 3b).

To further confirm that the radiomic feature-based classifier had a similar prognostic value, we applied it to the whole cohort of 199 patients with NPC, classifying 75 (37.7%) patients as high risk, and 124 (62.3%) as low risk. 5-year disease progression-free survival was 28.0% (95% CI 19.5–40.3) for the high-risk group and 47.6% (40.0–57.2) for the low-risk group (HR 1.73, 95% CI 1.21–2.46; P < 0.003; Fig. 3c).

For clinical factors, the results of multivariable Cox regression analysis showed that N stage (P = 0.004) and histology (P = 0.027) were significantly associated with disease progression but not for other clinical variables (P > 0.05) (Table 3). Rad-score (P = 0.04), N stage (P < 0.001), and histology (P = 0.04) were independent prognostic factors of PFS in patients with NPC (Table 4).

**Table 3:** Multivariable Cox regression analysis of clinical risk factors of disease progression in the training cohort

Variable	β	SE	z	*P*	HR	95%CI	
						Lower	Upper
Gender	− 0.26	0.28	− 0.92	0.34	0.77	0.45	1.33
Age	< 0.01	0.01	0.08	0.94	1.00	0.98	1.02
Histology	− 0.47	0.21	− 2.22	0.03^*^	0.62	0.41	0.95
EGFR	− 0.25	0.27	− 0.94	0.35	0.78	0.46	1.32
VEGF	− 0.46	0.27	− 1.72	0.09	0.63	0.37	1.07
T stage	0.21	0.16	1.32	0.19	1.23	0.90	1.69
N stage	0.47	0.17	2.91	< 0.01^*^	1.63	1.17	2.26
Overall stage	0.04	0.26	0.16	0.87	1.04	0.62	1.74

*SE* standard error, *HR* hazard ratio, *EGFR* epidermal growth factor receptor, *VEGF* vascular endothelial growth factor, *CI* confidence interval, * indicates significant difference

**Table 4:** Multivariable Cox regression analysis of risk factors of disease progression in the training cohort

Variable	β	SE	z	*P*	HR	95%CI	
						Lower	Upper
Rad-Score	2.63	1.304	2.02	0.04*	13.88	1.078	178.637
N stage	0.45	0.12	3.69	< 0.01*	1.57	1.24	1.99
Histology	− 0.44	0.21	2.10	0.04*	0.65	0.43	0.97

*SE* standard error, *HR* hazard ratio, *CI* confidence interval, * indicates significant difference

**Fig. 4:**
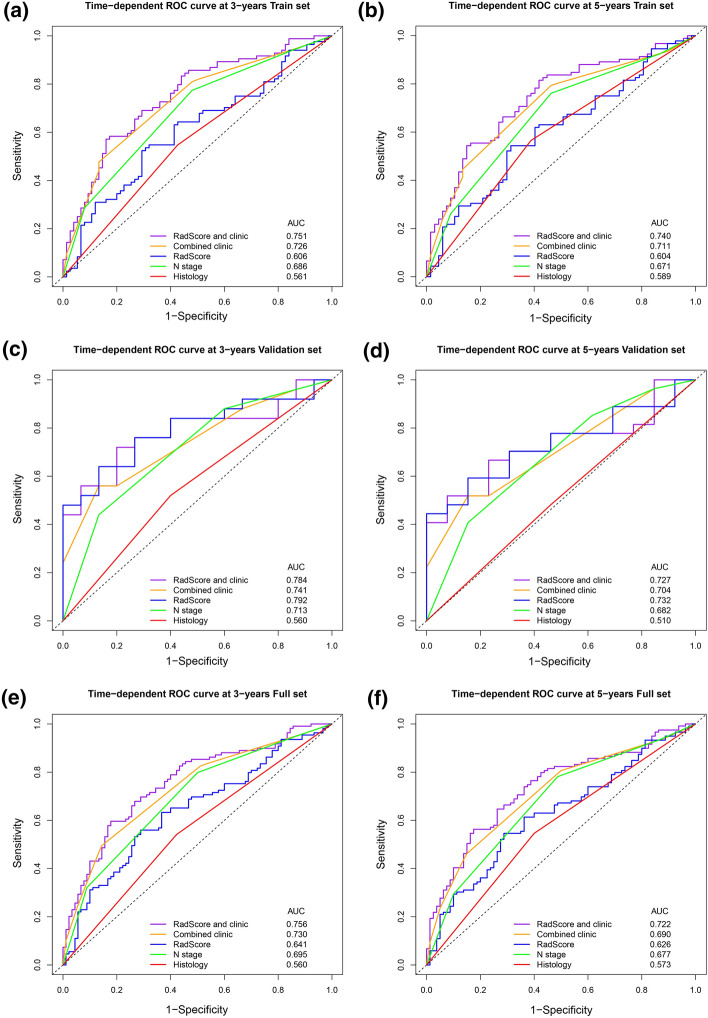
Time-dependent ROC curves compare the prognostic accuracy of the Rad-Score with clinicopathological risk factors in the training set (**a**, **b**), validation set (**c**, **d**), and the combined set (**e**, **f**) with NPC of 3-year and 5-year disease PFS

The Rad-Score combined with clinicopathological risk factor (N stage and histology) achieved higher AUC than any clinicopathological risk factor for prediction of 3-year and 5-year disease PFS (Fig. 4), although there was no statistical difference in the validation set at 3-year disease PFS (P = 0.18, 0.06, 0.26) and 5-year disease PFS (P = 0.46, 0.07, 0.58) compared with N stage, histology, and two clinicopathological risk factors combined, respectively.

**Fig. 5:**
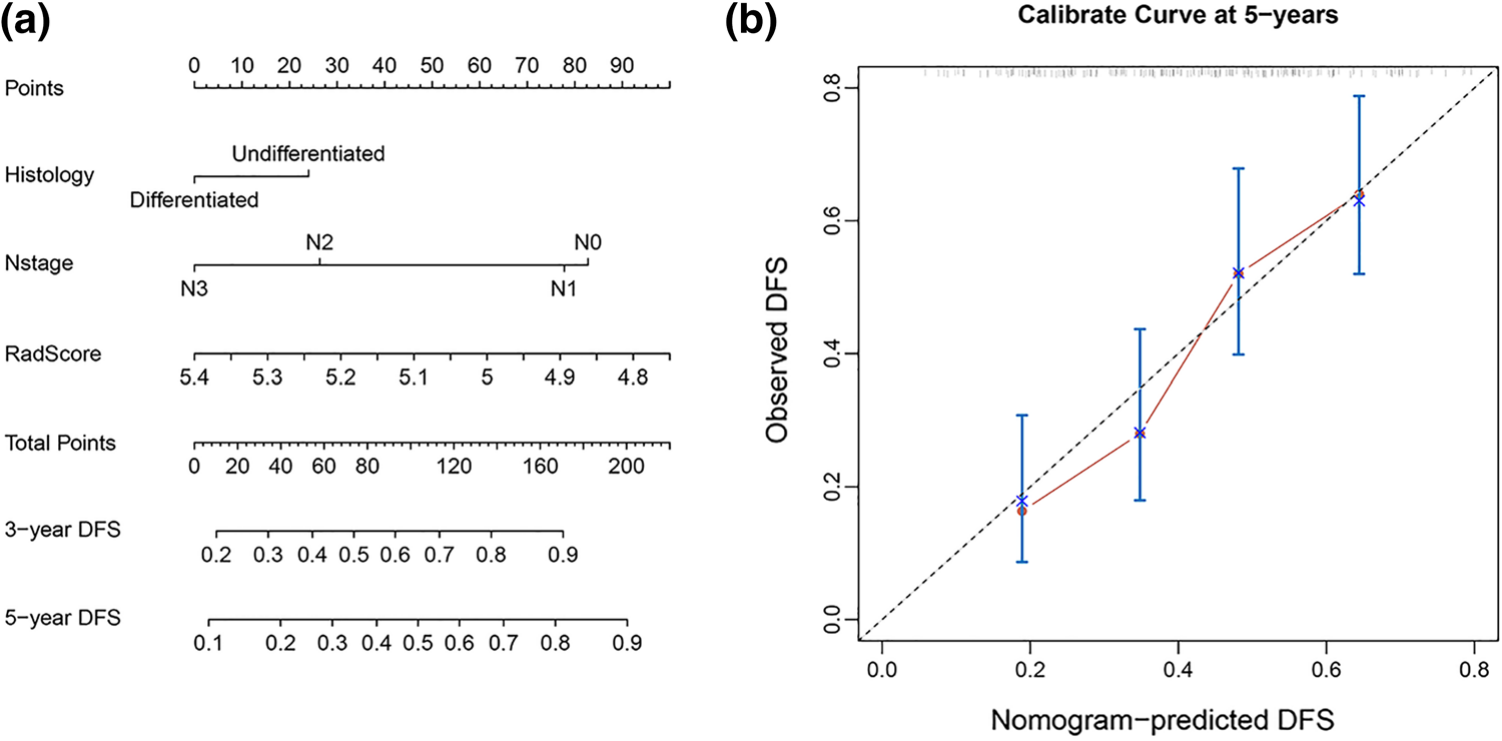
**a** Nomogram to predict risk of 5-year disease progression-free survival with non-distant metastatic NPC after tumor remission. **b** Plots depict the calibration of the model in terms of agreement between predicted and observed 5-year outcomes

To provide the clinician with a quantitative method for predicting the risk of disease progression in NPC patients, we constructed a nomogram integrating Rad-Score and two clinic features (Fig. 5a). Good calibration was observed for the probability of disease progression (Fig. 5b).

## Discussion

In this study, we developed and validated a novel prognostic tool, Rad-Score, based on one radiomic feature to improve the prediction of disease progression for patients with NPC after pathological complete remission. Our results showed that this tool can successfully categorize patients into high-risk and low-risk groups with large differences in 5-year disease progression-free survival. And the nomogram showed that Rad-Score can add prognostic value to clinicopathological prognostic features.

As we know, radiomics is hypothesized to illustrate the histological heterogeneity of tumors [4]. However, most of the current studies do not mention whether the radiomic features extracted from tumors are different from normal tissue. We performed a Wilcoxon signed-rank test based on the radiomic features extracted from tumors and normal masticatory muscles of 37 patients for feature selection. The next radiomic analysis was based on radiomic features that have significant statistical differences between tumors and normal tissues. Zhu et al. [17] first extracted 1,027 radiomic features with significant statistical differences from 1,452 radiomics features by analysis of variance (ANOVA) for predicting the risk of local recurrence in NPC, but features extracted from CT images of locally recurrent and non-recurrent patients. The use of radiomic features differentially expressed between tumors and normal tissue allowed the analysis more specific to our study and provided a new direction for research. However, it remains to be further explored which method may have greater prognostic accuracy compared to all the radiomics features included in the analysis.

CET1-w_GLDM_Dependence-Entropy was the only disease progression-related radiomic feature with significance found in the present study, which is extracted from CET1-w images and considered to reflect intratumor heterogeneity from the perspective of grayscale arrangement [18]. Entropy is inversely proportional to uniformity, representing the irregularity of pixel intensity values in an image, which reflects increased heterogeneity [19]. In our study, higher CET1-w_GLDM_Dependence-Entropy was a predictive factor for disease progression in patients with NPC; heterogeneous enhancement within a tumor on CE-T1WI was associated with poor prognosis. Similar results were also found in other cancers. For example, rectal cancer with more heterogeneity on less entropy is associated with poor OS and PFS [20]. Therefore, our Rad-Score based on CET1-w_GLDM_Dependence-Entropy for patients with NPC is a prognostic and predictive method, in that patients with a high value have a greater likelihood of disease progression. After Pearson correlation analysis, all the four radiomics features included in multivariate Cox regression analysis were derived from the CE-T1WI sequence. The results may be explained that the quantitative image features extracted from the CET1-w images are more likely to reflect the intra-tumor heterogeneity and architecture (e.g., tumor angiogenesis) [21]. More homogeneity on contrast-enhanced radiographic images suggests more homogeneous angiogenesis within the tumor, associated with better prognosis in head and neck squamous cell carcinoma [22, 23].

Another difference between the present study and the previous studies was that we analyzed the stability of VOIs segmentation by calculating the DSC. Different from the ICC calculation for radiomic features [24], DSC analyzes the 3D consistency for the segmentation of the VOIs with NPC [25], for which we could select stable and robust features that are not affected by variability in segmentation.

Some limitations of the present study need to be addressed. This is retrospective, with limited generalizability as all patients come from a single-center, and the distribution of clinical characteristics might be different in other areas, making it susceptible to the inherent biases of such a study format. Our results should be further validated by prospective studies in multicenter clinical trials. We only focused on T2WI/FS and CE-T1WI sequences, functional imaging techniques such as dynamic contrast-enhanced (DCE), diffusion-weighted imaging (DWI) could be considered in future studies. It should be noted that we did not investigate the association between the plasma EBV DNA copy number and disease progression in this study. Although this factor has been confirmed as a potentially useful predictor for the prognosis of NPC, this information was not available for most of our cohort due to the longer visits. Another shortcoming of our research was that NPC patients were treated by concurrent radiotherapy and chemotherapy or radiotherapy alone, with or without targeted therapy, using varying doses of radiotherapy regimen. Although patients’ treatment was based on TNM staging and National Comprehensive Cancer Network guidelines, these different strategies might be confounding factors in evaluating PFS.

In conclusion, our findings show that the identified radiomics signature has the potential to be used as a biomarker for risk stratification for DFS in patients NPC. As a non-invasive MR-based imaging biomarker, the radiomics-based classifier may provide a valuable and practical method to identify individual characteristics to guide personalized therapeutic regimen selection for patients with NPC.

## Supplementary Information

**Additional file 1.**

**Additional file 2.**

**Additional file 3.**

**Additional file 4.**

**Additional file 5.**

**Additional file 6.**

**Additional file 7.**

## Data Availability

Data are available in the Article and Supplementary Information. All other data can be provided upon reasonable request to the corresponding author.

## Abbreviations

AUC: Area under the curve
CE-T1WI: Contrast-enhanced T1-weighted sequence
CI: Confidence interval
DSC: Dice similarity coefficient
EBV: Epstein-Barr virus
EGFR: Epidermal growth factor receptor
PFS: Progression-free survival
HR: Hazard ratio
LASSO: Least absolute shrinkage and selection operator
NPC: Nasopharyngeal carcinoma
ROC: Receiver operating characteristic
TNM: Tumor-node-metastasis
T2WI/FS: T2-weighted fat-suppressed sequence
VEGF: Vascular endothelial growth factor
VOI: Volume of interest

## Code availability

The code of the proposed method can be provided upon reasonable request to the corresponding authors.

## References

[1] Bray F, Ferlay J, Soerjomataram I, Siegel RL, Torre LA, Jemal A (2018). Global cancer statistics 2018: GLOBOCAN estimates of incidence and mortality worldwide for 36 cancers in 185 countries. CA Cancer J Clin.

[2] Chan A, Hui EP, Ngan R (2018). Analysis of plasma Epstein-Barr virus DNA in nasopharyngeal cancer after chemoradiation to identify high-risk patients for adjuvant chemotherapy: a randomized controlled trial. J Clin Oncol.

[3] Qu W, Li S, Zhang M, Qiao Q (2017). Pattern and prognosis of distant metastases in nasopharyngeal carcinoma: a large-population retrospective analysis. Cancer Med.

[4] Mayerhoefer ME, Materka A, Langs G (2020). Introduction to radiomics. J Nucl Med.

[5] Liang ZG, Tan HQ, Zhang F (2019). Comparison of radiomics tools for image analyses and clinical prediction in nasopharyngeal carcinoma. Br J Radiol.

[6] Zhao L, Gong J, Xi Y (2020). MRI-based radiomics nomogram may predict the response to induction chemotherapy and survival in locally advanced nasopharyngeal carcinoma. Eur Radiol.

[7] Mao J, Fang J, Duan X (2019). Predictive value of pretreatment MRI texture analysis in patients with primary nasopharyngeal carcinoma. Eur Radiol.

[8] Du R, Lee VH, Yuan H (2019). Radiomics model to predict early progression of nonmetastatic nasopharyngeal carcinoma after intensity modulation radiation therapy: a multicenter study. Radiol Artif Intell.

[9] Pan JJ, Ng WT, Zong JF (2016). Proposal for the 8^th^ edition of the AJCC/UICC staging system for nasopharyngeal cancer in the era of intensity-modulated radiotherapy. Cancer.

[10] Duane F, Aznar MC, Bartlett F (2017). A cardiac contouring atlas for radiotherapy. Radiother Oncol.

[11] Liu MZ, Ge YQ, Li MR, Wei W (2021). Prediction of BRCA gene mutation status in epithelial ovarian cancer by radiomics models based on 2D and 3D CT images. BMC Med Imag.

[12] Arefan D, Chai RM, Sun M, Zuley ML, Wu SD (2020). Machine learning prediction of axillary lymph node metastasis in breast cancer: 2D versus 3D radiomic features. Med Phys.

[13] Maritz JS (1985). Models and the use of signed rank tests. Stat Med.

[14] Tibshirani R (1997). The lasso method for variable selection in the Cox model. Stat Med.

[15] Gui J, Li H (2005). Penalized Cox regression analysis in the high-dimensional and low-sample size settings, with applications to microarray gene expression data. Bioinformatics.

[16] Schober P, Boer C, Schwarte LA (2018). Correlation coefficients: appropriate use and interpretation. Anesth Analg.

[17] Zhu C, Huang H, Liu X (2021). A clinical-radiomics nomogram based on computed tomography for predicting risk of local recurrence after radiotherapy in nasopharyngeal carcinoma. Front Oncol.

[18] Fan TW, Malhi H, Varghese B (2019). Computed tomography-based texture analysis of bladder cancer: Differentiating urothelial carcinoma from micropapillary carcinoma. Abdom Radiol (NY).

[19] Brown AL, Jeong J, Wahab RA, Zhang B, Mahoney MC (2021). Diagnostic accuracy of MRI textural analysis in the classification of breast tumors. Clin Imaging.

[20] Hotta M, Minamimoto R, Gohda Y (2021). Prognostic value of (18)F-FDG PET/CT with texture analysis in patients with rectal cancer treated by surgery. Ann Nucl Med.

[21] Ibrahim MA, Dublin AB (2018). Magnetic Resonance Imaging (MRI), Gadolinium.

[22] Barresi V, Cerasoli S, Vitarelli E, Tuccari G (2007). Density of microvessels positive for CD105 (endoglin) is related to prognosis in meningiomas. Acta Neuropathol.

[23] Bentzen SM, Atasoy BM, Daley FM (2005). Epidermal growth factor receptor expression in pretreatment biopsies from head and neck squamous cell carcinoma as a predictive factor for a benefit from accelerated radiation therapy in a randomized controlled trial. J Clin Oncol.

[24] Shen H, Wang Y, Liu D (2020). Predicting Progression-Free survival using MRI-Based radiomics for patients with nonmetastatic nasopharyngeal carcinoma. Front Oncol.

[25] Tanabe Y, Ishida T, Eto H, Sera T, Emoto Y (2019). Evaluation of the correlation between prostatic displacement and rectal deformation using the Dice similarity coefficient of the rectum. Med Dosim.

