# Supplementary material for: Prognostic and predictive value of radiomics features at MRI in nasopharyngeal carcinoma
Source: Discov Oncol. 2021 Dec 17;12:63. doi: 10.1007/s12672-021-00460-3 (PMC8683387; doi:10.1007/s12672-021-00460-3)
Supplement: Supplementary file 1 — Additional file 1. [file 12672_2021_460_MOESM1_ESM.pdf]

Table 1 Details in parameters used to acquire the imaging data.

MR scan（Sequence）	3.0T MR scanner (GE Discovery MR 750, General Electric Medical Systems)	
	T2WI/FS	CE-T1WI
TR (ms)	8472	315
TE (ms)	85	2.9
FOV (cm)	24	24
Slice thickness（mm）	4.0	4.0
Space between the slices（mm）	0.4	0.4
Bandwidth（kHz）	50	31.25
ETL	17	21
NEX	2	1
Matrix	320×256	320×256

TR, repetition time (ms); TE, echo time (ms); FOV, field of view (cm); ETL, echo train length; NEX, number of excitations; T2WI, T2-weighted imaging; FS, frequency saturation; CE, contrast-enhanced; T1WI, T1-weighted imaging.

Prognostic and predictive value of radiomics features at MRI in nasopharyngeal carcinoma.

Discover Oncology.

Dan Bao; Yanfeng Zhao; Zhou Liu; Hongxia Zhong; Yayuan Geng; Meng Lin; Lin Li; Xinming Zhao; Dehong Luo.
The corresponding author: Dehong Luo, e-mail address: pumccancer@163.com,

Department of Radiology, National Cancer Center/National Clinical Research Center for Cancer/Cancer Hospital, Chinese Academy of Medical Sciences and Peking Union Medical College, Beijing, 100021, China.
