# Supplementary figures and images for: Prognostic and predictive value of radiomics features at MRI in nasopharyngeal carcinoma
Source: Discov Oncol. 2021 Dec 17;12:63. doi: 10.1007/s12672-021-00460-3 (PMC8683387; doi:10.1007/s12672-021-00460-3)

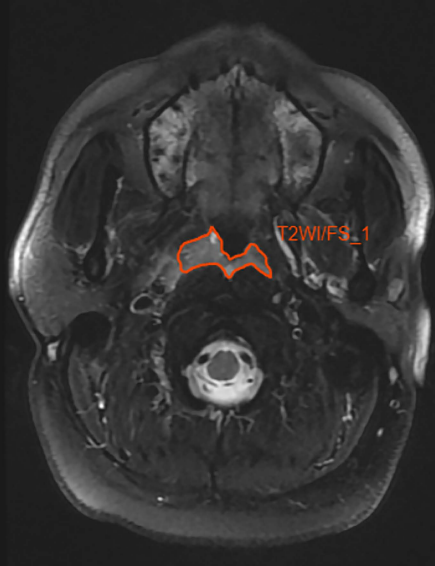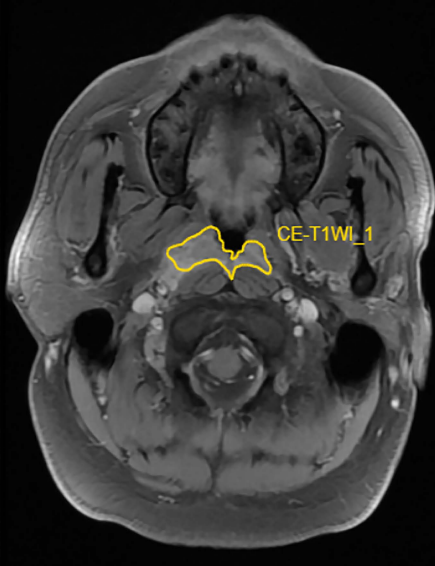

Supplement: Supplementary file 2 — Additional file 2. [file 12672_2021_460_MOESM2_ESM.pdf]
