# Supplementary material for: Prognostic and predictive value of radiomics features at MRI in nasopharyngeal carcinoma
Source: Discov Oncol. 2021 Dec 17;12:63. doi: 10.1007/s12672-021-00460-3 (PMC8683387; doi:10.1007/s12672-021-00460-3)
Supplement: Supplementary file 3 — Additional file 3. [file 12672_2021_460_MOESM3_ESM.pdf]

imageType:
    Original: {}
    LoG:
        sigma: [2.0, 3.0, 4.0, 5.0]
    Logarithm: {}
    Exponential: {}
    Gradient: {}
    Square: {}
    SquareRoot: {}
    LocalBinaryPattern2D: {}
    Wavelet- LHL: {}
    Wavelet- LHH: {}
    Wavelet- HLL: {}
    Wavelet- LLH: {}
    Wavelet- HLH: {}
    Wavelet- HHH: {}
    Wavelet- HHL: {}
    Wavelet- LLL: {}

featureClass:
    shape:
    firstorder:
    glcm:
    glrlm:
    glszm:
    gldm:
    ngtdm:

setting:
    normalize: true
    normalizeScale: 1
    interpolator: 'sitkBSpline'
    resampledPixelSpacing: [1, 1, 1]
    binWidth: 25
    voxelArrayShift: 300
    label: 1

Prognostic and predictive value of radiomics features at MRI in nasopharyngeal carcinoma.

Discover Oncology.

Dan Bao; Yanfeng Zhao; Zhou Liu; Hongxia Zhong; Yayuan Geng; Meng Lin; Lin Li; Xinming Zhao; Dehong Luo.

The corresponding author: Dehong Luo, e-mail address: pumccancer@163.com, Department of Radiology, National Cancer Center/National Clinical Research Center for Cancer/Cancer Hospital, Chinese Academy of Medical Sciences and Peking Union Medical College, Beijing,

100021, China.
