# Supplementary material for: Prognostic and predictive value of radiomics features at MRI in nasopharyngeal carcinoma
Source: Discov Oncol. 2021 Dec 17;12:63. doi: 10.1007/s12672-021-00460-3 (PMC8683387; doi:10.1007/s12672-021-00460-3)
Supplement: Supplementary file 4 — Additional file 4. [file 12672_2021_460_MOESM4_ESM.pdf]

Table2 Classification of 1409 radiomic features.

Image type		Feature class						
		First-order	Shape	GLCM	GLDM	GLRLM	GLSZM	NGTDM
Original		18	14	24	14	16	16	5
Logarithm		18		24	14	16	16	5
Exponential		18		24	14	16	16	5
Gradient		18		24	14	16	16	5
Square		18		24	14	16	16	5
SquareRoot		18		24	14	16	16	5
LocalBinaryPattern2D		18		24	14	16	16	5
Wavelet-	LHL	18		24	14	16	16	5
	LHH	18		24	14	16	16	5
	HLL	18		24	14	16	16	5
	LLH	18		24	14	16	16	5
	HLH	18		24	14	16	16	5
	HHH	18		24	14	16	16	5
	HHL	18		24	14	16	16	5
	LLL	18		24	14	16	16	5

GLCM gray level co-occurrence matrix, *GLDM* gray level dependence matrix, *GLRLM* gray level run length matrix, *GLSZM* gray level size zone matrix, *NGTDM* neighbouring gray tone difference matrix

Prognostic and predictive value of radiomics features at MRI in nasopharyngeal carcinoma.

Discover Oncology.

Dan Bao; Yanfeng Zhao; Zhou Liu; Hongxia Zhong; Yayuan Geng; Meng Lin; Lin Li; Xinming Zhao; Dehong Luo.

The corresponding author: Dehong Luo, e-mail address: pumccancer@163.com, Department of Radiology, National Cancer Center/National Clinical Research Center for Cancer/Cancer Hospital, Chinese Academy of Medical Sciences and Peking Union Medical College, Beijing, 100021, China.
