# Supplementary material for: Prognostic and predictive value of radiomics features at MRI in nasopharyngeal carcinoma
Source: Discov Oncol. 2021 Dec 17;12:63. doi: 10.1007/s12672-021-00460-3 (PMC8683387; doi:10.1007/s12672-021-00460-3)
Supplement: Supplementary file 5 — Additional file 5. [file 12672_2021_460_MOESM5_ESM.pdf]

“survminer” package in R software

Link to the “survminer” package:

https://www.rdocumentation.org/packages/survminer/versions/0.4.9/topics/surv_cutpoint

Version:

survminer(version 0.4.9)

Function of the package:

Determine the optimal cut-point for one or multiple continuous variables at once, using the maximally selected rank statistics from the 'maxstat' R package. This is an outcome-oriented methods providing a value of a cut-point that correspond to the most significant relation with outcome (here, survival);

surv_cutpoint(): Determine the optimal cut-point for each variable using 'maxstat'.

Usage:

surv_cutpoint(data, time = "time", event = "event", variables , minprop = 0.1, progressbar = TRUE)

Arguments:

data

a data frame containing survival information (time, event) and continuous variables (e.g.: gene expression data).

time, event

column names containing time and event data, respectively. Event values should be 0 or 1.

variables

a character vector containing the names of variables of interest, for which we want to estimate the optimal cut-point.

minprop

the minimal proportion of observations per group.

progressbar

logical value. If TRUE, show progress bar. Progressbar is shown only, when the number of variables > 5.

Prognostic and predictive value of radiomics features at MRI in nasopharyngeal carcinoma.

Discover Oncology.

Dan Bao; Yanfeng Zhao; Zhou Liu; Hongxia Zhong; Yayuan Geng; Meng Lin; Lin Li; Xinming Zhao; Dehong Luo.

The corresponding author: Dehong Luo, e-mail address: pumccancer@163.com,

Department of Radiology, National Cancer Center/National Clinical Research Center for Cancer/Cancer Hospital, Chinese Academy of Medical Sciences and Peking Union Medical College, Beijing, 100021, China.
