# Supplementary material for: Prognostic and predictive value of radiomics features at MRI in nasopharyngeal carcinoma
Source: Discov Oncol. 2021 Dec 17;12:63. doi: 10.1007/s12672-021-00460-3 (PMC8683387; doi:10.1007/s12672-021-00460-3)
Supplement: Supplementary file 6 — Additional file 6. [file 12672_2021_460_MOESM6_ESM.pdf]

“timeROC” package in R software

Link to the “timeROC” package:

https://www.rdocumentation.org/packages/timeROC/versions/0.4/topics/timeROC

Version:

timeROC (version 0.4)

timeROC: Time-dependent ROC curve estimation

Description:

Inverse Probability of Censoring Weighting (IPCW) estimation of Cumulative/Dynamic time-dependent ROC curve. The function works in the usual survival setting as well as in the competing risks setting. Computation of the iid-representation of areas under time-dependent ROC curves is implemented. This enables computation of inference procedures: Confidence intervals and tests for comparing two AUCs of two different markers measured on the same subjects.

Usage:

```
timeROC(T, delta, marker, other_markers = NULL, cause,
        weighting = "marginal", times, ROC = TRUE, iid = FALSE)
```

Arguments:

T

The vector of (censored) event-times.

delta

The vector of event indicators at the corresponding value of the vector **T**. Censored observations must be denoted by the value `0`.

marker

The vector of the marker values for which we want to compute the time-dependent ROC curves. Without loss of generality, the function assumes that larger values of the marker are associated with higher risks of events. If lower values of the marker are associated with higher risks of events, then reverse the association adding a minus to the marker values.

other_markers

A matrix that contains values of other markers that we want to take into account for computing the inverse probability of censoring weights. The different columns represent the

different markers. This argument is optional, and ignored if **method="marginal"**. Default value is **other_markers=NULL**.

cause

The value of the event indicator that represents the event of interest for which we aim to compute the time-dependent ROC curve. Without competing risks, it must be the value that indicates a non-censored obsevation (usually **1**). With competing risks, subjects can undergo different type of events; then, it must be the value corresponding to the event of interest, for which we aim to compute the ROC curve (usually **1** or **2**).

weighting

The method used to compute the weights. **weighting="marginal"** uses the Kaplan-Meier estimator of the censoring distribution. **weighting="cox"** and **weighting="aalen"** model the censoring by the Cox model and the additive Aalen model respectively. Default value is **weighting="marginal"**.

times

The vector of times points "t" at which we want to compute the time-dependent ROC curve. If vector **times** contains only a single value, then value zero is added.

ROC

A logical value that indicates if we want to save the estimates of sensitivities and specificities. Default value is **ROC = TRUE**.

iid

A logical value that indicates if we want to compute the iid-representation of the area under time-dependent ROC curve estimator. **iid = TRUE** is required for computation of all inference procedures (Confidence intervals or test for comparing AUCs). For large sample size (greater than 2000, say) and/or large length of vector times, the computation of the iid representations might be time-consuming. Default value is **iid = FALSE**.

Details

This function computes Inverse Probability of Censoring Weighting (IPCW) estimates of Cumulative/Dynamic time-dependent ROC curve. By definition, time-dependent ROC curve intrinsically depends on the definitions of time-dependent cases and controls.

Let T_i denote the event time of the subject i.

Without competing risks : A case is defined as a subject *i* with $T_i \leqslant t$. A control is defined as a subject *i* with $Ti > t$.

With competing risks : In this setting, subjects may undergo different type of events, denoted by δ_i in the following. Let suppose that we are interested in the event $\delta_i = 1$. Then, a case is defined as a subject *i* with $T_i \leqslant t$ and $\delta_i = 1$. With competing risks, two definitions of controls were suggested: **(i)** a control is defined as a subject *i* that is free of any event, i.e with $T_i > t$, and (ii) a control is defined as a subject *i* that is not a case, i.e with $T_i > t$ or with $T_i \leqslant t$ and $\delta_i \neq 1$. For all outputs of this package, objects named with _1 refer to definition **(i)**. For instance AUC_1 or se_1 refer to time-dependent area under the ROC curve and its estimated standard error according to the definition **(i)**. Objects named with _2 refer to definition **(ii)**.

Value

Object of class "ipcwsurvivalROC" or "ipcwcompetingrisksROC", depending on if there is competing risk or not, that is a list. For these classes, there are print, plot and confint methods. Most objects that they contain are similar, but some are specific to each class.

Specific objects of class "ipcwsurvivalROC":

- AUC : vector of time-dependent AUC estimates at each time points.
- TP : matrix of time-dependent True Positive fraction (sensitivity) estimates.
- FP : matrix of time-dependent False Positive fraction (1-specificity) estimates.

Specific objects of class "ipcwcompetingrisksROC":

- AUC_1 : vector of time-dependent AUC estimates at each time points with definition **(i)** of controls (see Details).
- AUC_2 : vector of time-dependent AUC estimates at each time points with definition **(ii)** of controls (see Details).
- TP : matrix of time-dependent True Positive fraction (sensitivity) estimates.
- FP_1 : matrix of time-dependent False Positive fraction (1-specificity) estimates with definition **(i)** of controls (see Details).
- FP_2 : matrix of time-dependent False Positive fraction (1-specificity) estimates with definition **(ii)** of controls (see Details).

Objects common to both classes:

- times : the time points for which the time-dependent ROC curves were computed.

• weights : a object of class "IPCW", containing all informations about the weights.

• computation_time : the total computation time.

• CumulativeIncidence : the vector of estimated probabilities of being a case at each time points.

• survProb : the vector of estimated probabilities of being event-free at each time points.

• Stats : a matrix containing descriptive statistics at each time points (like numbers of observed
cases or censored observations before each time points).

• iid : the logical value of parameter iid used in argument.

• n : the sample size, after having omitted missing vaues.

• inference : a list that contains, among other things, iid-representations and estimated standard
errors of the estimators, and that is used for computation of comparison tests and confidence intervals.

• computation_time : the computation time, in seconds.

References

Hung, H. and Chiang, C. (2010). Estimation methods for time-dependent AUC with survival data. Canadian Journal of Statistics, 38(1):8-26

Uno, H., Cai, T., Tian, L. and Wei, L. (2007). Evaluating prediction rules for t-years survivors with censored regression models. Journal of the American Statistical Association, 102(478):527-537.

Blanche, P., Dartigues, J. F., & Jacqmin-Gadda, H. (2013). Estimating and comparing timedependent areas under receiver operating characteristic curves for censored event times with competing risks. Statistics in medicine, 32(30), 5381-5397.

P. Blanche, A. Latouche, V. Viallon (2013). Time-dependent AUC with right-censored data: A Survey. Risk Assessment and Evaluation of Predictions, 239-251, Springer, http://arxiv.org/abs/1210.6805.

Prognostic and predictive value of radiomics features at MRI in nasopharyngeal carcinoma. Discover Oncology.

Dan Bao; Yanfeng Zhao; Zhou Liu; Hongxia Zhong; Yayuan Geng; Meng Lin; Lin Li; Xinming Zhao; Dehong Luo.

The corresponding author: Dehong Luo, e-mail address: pumccancer@163.com,

Department of Radiology, National Cancer Center/National Clinical Research Center for Cancer/Cancer Hospital, Chinese Academy of Medical Sciences and Peking Union Medical College, Beijing, 100021, China.
