# Supplementary material for: Prognostic and predictive value of radiomics features at MRI in nasopharyngeal carcinoma
Source: Discov Oncol. 2021 Dec 17;12:63. doi: 10.1007/s12672-021-00460-3 (PMC8683387; doi:10.1007/s12672-021-00460-3)
Supplement: Supplementary file 7 — Additional file 7. [file 12672_2021_460_MOESM7_ESM.pdf]

Table3 The R packages used for statistical analysis

R package	statistical analysis
glmnet	LASSO Cox regression
Survival; survminer	multivariable Cox regression analysis; log-rank test
timeROC	time-dependent ROC curve analysis
ggplot2	Kaplan-Meier
rms	nomogram construction and calibration

LASSO least absolute shrinkage and selection operator, *ROC* receiver operating characteristic

Prognostic and predictive value of radiomics features at MRI in nasopharyngeal carcinoma.

Discover Oncology.

Dan Bao; Yanfeng Zhao; Zhou Liu; Hongxia Zhong; Yayuan Geng; Meng Lin; Lin Li; Xinming Zhao; Dehong Luo.

The corresponding author: Dehong Luo, e-mail address: pumccancer@163.com, Department of Radiology, National Cancer Center/National Clinical Research Center for Cancer/Cancer Hospital, Chinese Academy of Medical Sciences and Peking Union Medical College, Beijing, 100021, China.
